# Is Alum Poisonous?

**Published:** 1879-08

**Authors:** 


### *IS ALUM POISONOUS?*

This question has caused a good deal of discussion. Alum is used by many bakers to whiten their bread, enabling them to use an inferior flour. It is more extensively employed as a cheap substitute for cream of tartar, in the manufacture of baking powders. It has not been considered immediately dangerous; although if continued it induces dyspepsia and obstinate constipation. But the fact that many cases of poisoning have occurred from baking powders which contained alum, puts the question in a more serious aspect, and prudent people will exercise caution in the selection of baking powders.

Under what conditions, then, does this substance—formerly used only for mechanical or medicinal purposes—become poisonous? They are certainly obscure, and at present we can only surmise what *they may be.* We suspect that the cause exists in the individual poisoned; some peculiarity of the constitution producing a morbid change in the secretions of the stomach, with which the alum combines and forms an active poison; or the secretions may be healthy but in unusual proportions, and that these less or greater proportions, in combination with the alum, constitute a poison.

For example: two parts of mercury and two parts of chlorine form calomel, which is not poisonous; but change the proportions to one part of mercury and two parts of chlorine, and we get corrosive sublimate, which is a deadly poison.

Then, again, we know nothing of the causes of constitutional peculiarities. Why is it that one person can eat all kinds of green fruits and vegetables with impunity, while the same course might cost another individual his life? One person can handle poison ivy and sumac without being in the least affected, another is poisoned if he approaches to within ten feet of them. Out of a family residing in a malarial district, some of its members will suffer half the year with fever and ague, while the others will enjoy excellent health during the entire year. Foods that are wholesome to some persons are actually poisonous to others. This is especially true of some kinds of fish. There is no safety in taking alum into the stomach, as it is shown to be always injurious, and often dangerous. Baking powders properly compounded, and containing pure cream of tartar instead of alum, are more convenient than yeast; and bread and pastry made with them are just as wholesome, and far more palatable. We are in entire sympathy with the "Royal Baking Powder Company," which commenced and is vigorously conducting this war against the use of alum in baking powders, and they deserve the gratitude of the community whom they are endeavoring to protect.

Will not some prominent manufacturer of pure candies follow their example, and expose the secrets of a business that is doing untold mischief to little children?

